# Prevalence and risk factors of intracranial and extracranial artery stenosis in asymptomatic rural residents of 13 villages in China

**DOI:** 10.1186/s12883-017-0924-0

**Published:** 2017-07-18

**Authors:** Haiqiang Jin, Qing Peng, Ding Nan, Pu Lv, Ran Liu, Wei Sun, Yuming Teng, Yuanyuan Liu, Chenghe Fan, Haiying Xing, Ke Xu, Yining Huang

**Affiliations:** Department of Neurology, Peking University First Hospital, No.8 Xishiku Street, Xicheng District, Beijing, 100034 People’s Republic of China

**Keywords:** Extracranial stenosis, Intracranial stenosis, Prevalence, Risk factor

## Abstract

**Background:**

The present study aimed to investigate the prevalence and risk factors for extracranial carotid artery stenosis (ECAS) and intracranial carotid artery stenosis (ICAS) simultaneously in asymptomatic Chinese pure rural population.

**Methods:**

We analyzed 2589 asymptomatic subjects aged over 30 yr. by ultrasonography and transcranial Doppler simultaneously in 13 isolated villages by door-to-door investigation. Both ECAS and ICAS were defined as more than 50% stenosis. Demographics, medical history documentation, and investigation of biochemical results were performed for each subject. Univariate and multivariate logistic regression analyses were employed to assess the risk factors associated with ECAS and ICAS, respectively.

**Results:**

One hundred twenty-two (4.7%) residents with ICAS and 56 (2.2%) with ECAS were found in 2589 subjects. Three factors emerged as independent risk factors for ICAS: age (95% confidence interval [CI] = 1.01–1.04, odds ratio [OR] = 1.07), hypertension (95% CI = 1.98–4.37, OR = 2.94), and diabetes mellitus (95% CI = 1.72–4.38, OR = 2.75). As for ECAS, five factors presented as independent risk factors: age (95% CI = 1.09–1.11, OR = 1.10), male sex (95% CI = 1.01–1.02, OR = 1.01), diabetes mellitus (95% CI = 1.10–2.12, OR = 1.53), systolic blood pressure (95% CI = 1.95–2.88, OR = 2.37), and total cholesterol (95% CI = 1.00–1.13, OR = 1.06).

**Conclusions:**

ICAS and ECAS were relatively common among asymptomatic rural Chinese subjects. Although they shared similar risk factors, differences still existed between them.

## Background

Atherosclerosis is the leading cause of stroke. Since atherosclerotic lesions develop silently over a time span of years until they become symptomatic, identification of latent cerebral atherosclerotic disease markers is potentially useful in the setting of primary prevention of stroke and interventions [1]. In general, the cerebral atherosclerotic disease classified as extracranial carotid artery stenosis (ECAS) and intracranial carotid artery stenosis (ICAS). ECAS refers to atherosclerotic narrowing of extracranial carotid arteries, specifically, the internal carotid arteries or the common and internal carotid arteries. Asymptomatic ECAS is commonly encountered in clinical practice. Its prevalence ranges from 0.1 to 7.5% in the general population and is highest in older men [2]. ICAS is the process of atherosclerosis that affects the intracranial large arteries, which is considered a major cause of stroke in blacks, Asians, and Hispanics, and a relatively infrequent cause of ischemic stroke in whites [3, 4]. Among symptomatic patients, the prevalence of intracranial atherosclerotic disease in the Caucasian population is around 10% [5], and 33–54% in Asian patients depending on race ethnicity [6]. The reason for the disparity is unknown. Moreover, concurrent stenosis of extracranial and intracranial vessels is common among Asians, with an incidence ranging from 10% to 48% in patients with symptomatic cerebrovascular diseases who are at high risk of further vascular events or death [7].

Previous studies are mostly hospital-based studies focusing on the risk factors for symptomatic ICAS or ECAS patients [5, 6, 8]. Due to the huge difference in rural and urban lifestyles as well as the geographic discrepancy in China, the prevalence and the role of classic risk factors for both asymptomatic ECAS and ICAS may also be different. Therefore, we performed population-based epidemiological study to inspect the prevalence and rick factors of ECAS and ICAS in a pure rural population of North China who lived there for generations and were close in lifestyle. ICAS was assessed by transcranial Doppler ultrasonography (TCD), which is now accepted as a sensitive and specific tool in the evaluation of ICAS [9], while ECAS was examined by carotid ultrasonography [10]. The residents received the TCD and carotid ultrasonography through door-to-door investigations.

## Methods

### Subjects

We randomly selected a sample of 3000 residents from 13 isolated rural villages in Sanhe County, Hebei Province, located in North China, ranging from 30 to 90 years old. Written informed consent was granted by all participants. We collected the demographic, anthropometric, and medical history of patients via questionnaire, and then conducted TCD examination, carotid artery ultrasound, and biochemistry investigation on each individual. Two hundred five subjects with poor acoustic temporal windows were excluded to avoid unreliable results from TCD. Two hundred six subjects who had a previous history of stroke or transient ischemic attack or coronary artery disease were also excluded. Ultimately, therefore, 2589 participants were eligible for analysis. Our study protocol was approved by the Ethics Committee of Peking University First Hospital.

Hypertension (HTN) was defined as repeated blood pressure > 140/90 mmHg or the need for chronic antihypertensive medication; DM was defined as fasting blood glucose >7.0 mmol/L, postprandial blood glucose >11.1 mmol/L, or the need for chronic hypoglycemic medications; smoking was defined as self-reported current or past habit of smoking; and alcohol use was defined as self-reported current or past habit of drinking alcohol.

### Transcranial Doppler ultrasonography examination

We selected TCD to measure the intracranial arterial stenosis [11]. The TCD examination was conducted by two experienced physicians using a portable device (EME Companion; Nicolet). The diagnostic criteria of ICAS were established in line with peak systolic flow velocity (PSV) criteria: >140 cm/s for the MCA; >120 cm/s for the ACA; >100 cm/s for PCA vertebral artery (VA), and basilar artery (BA); and >120 cm/s for the internal carotid siphon. In addition to the PSV criteria, the disturbance of the echo frequency, turbulence, or abnormal blood flow was also taken into consideration for ICAS diagnosis [12]. ICAS was diagnosed when one or more arteries showed evidence of stenosis. The terminals of the ICA, MCA, and ACA were defined as anterior circulation, while the VA, BA, and PCA were classified as posterior circulation.

### Carotid ultrasound measurements

Details of the carotid ultrasonographic examination methods have been previously described [10]. The sonography exam was also conducted by two experienced physicians using a high-resolution B-mode ultrasound machine with a 7.5-MHz transducer yielding an axial resolution of 0.1 mm. The regions from 30 mm proximal to the beginning of the dilation of the bifurcation bulb to 15 mm distal to the flow divider of both common carotid arteries (CCAs) were scanned. All measurements were conducted when scanning with the electronic caliper and were then recorded on photocopies. Intima media thickness (IMT) was defined as the mean of the thickness of intima media of the proximal and distal walls of the CCAs at the point of measurement. The thickened IMT was defined as IMT >0.9 mm. A plaque was defined as an area where IMT ≥1.5 mm. Furthermore, stenosis was defined as a condition whereby more than half of the lumen circumference of an artery was occupied by a plaque on a cross-sectional scan, which means the stenosis was ≥50% [13]. Color-flow Doppler examination was performed to confirm the diagnosis of stenosis.

### Biochemistry test

The blood samples of the residents were drawn after 8 h of fasting before the detection of TCD and carotid sonography and stored in a biobank at −80 °C. Blood glucose, total cholesterol, triglycerides, and creatinine were determined using a Beckman CX5 Automated Analyzer.

### Statistics

Continuous data were analyzed by *t* tests, while discrete data were processed by chi-square tests. For ICAS, residents were divided into two groups, an intracranial artery stenosis group and a group without intracranial artery stenosis, on the basis of the results of TCD. For ECAS, residents were assigned to two groups in accordance with the sonography: the normal, 0.9 < IMT < 1.5-mm and stenosis less than 50% group were merged to form the group without ECAS, and those with stenosis ≥50% formed the ECAS group. Variables found to be associated with intracranial or extracranial stenosis in the univariate analysis by *P* < 0.20 were further tested by backward stepwise, binary logistic regression to investigate the independent predictive factors for artery stenosis. To account for the effect of clustering at the village level, villages were introduced as a random effect in the final multivariate model. Statistical significance was defined as *P* < 0.05. The SPSS 16.0 (SPSS Inc., Chicago, IL, USA) software package was used for data analysis.

## Results

### Prevalence of cerebral atherosclerosis

All the subjects were of Han Chinese origin. Of the 2598 subjects, 1514 (58.3%) were female. The mean age of the residents was 55 with a maximum age of 94. For extracranial artery, 784 (30.2%) subjects had carotid plaques, and 56 (2.2%) had stenosis ≥50%. For intracranial artery, 122 (4.7%) were found of ICAS in 2598 individuals, with 80 (3.06%) involved with anterior circulation stenosis, 25 (0.96%) of posterior circulation stenosis, and 17 (0.65%) of both anterior and posterior circulation stenosis. For ICAS, the distribution of cerebral artery stenosis was shown in Table 1.

**Table 1:** Difference of ICAS and ECAS by gender

	Women	Men	*p* value
Cerebral atherosclerosis	1514	1084	0.06
Normal, n(%)	1421(93.9%)	1011(93.3%)	
ICAS only	68(4.5%)	42(3.9%)	
ECAS only	22(1.5%)	22(2.0%)	
ICAS and ECAS	3(0.2%)	9(0.8%)	
ECAS, n(%)	1514	1084	<0.001
Normal	999(66.0%)	528(48.7%)	
IMT	151(10.0%)	136(12.5%)	
Stenosis(<50%)	339(22.4%)	389(35.9%)	
Stenosis (≥50%)	25(1.7%)	31(2.9%)	
Distribution of ICAS, n(%)	1514	1084	0.99
None	1443(95.3%)	1033(95.3%)	
Anterior Circulation	47(3.1%)	33(3.0%)	
Posterior Circulation	14(0.9%)	11(1.0%)	
Anterior and posterior Circulation	10(0.7%)	7(0.6%)	

*ICAS* intracranial artery stenosis, *ECAS* extracranial artery stenosis, *IMT* intimal medial thickness

As for gender, the prevalence of ICAS and ECAS in men and women did not show a significant difference (*p* = 0.06); women had a slightly higher percentage (4.5% vs. 3.9%) of ICAS while men had a slightly higher percentage (2.0% vs. 1.5%) of ECAS. With respect to the degree of ECAS, there was a significant difference of prevalence between men and women (*p* < 0.001). Both stenosis <50% (35.9% vs. 22.4%) and ≥50% (2.9% vs. 1.7%) was more common in men than in women. Regarding the degree of ICAS, the prevalence in men and women was almost identical (*p* = 0.99).

### Risk factors for ICAS and ECAS

Regarding risk factors, 26.2% had hypertension and 7.6% had DM in the total 2589 subjects. Table 2 shows the univariate analysis of risk factors for ICAS and ECAS. Age, HTN, DM, systolic blood pressure (SBP), diastolic blood pressure (DBP), and fasting glucose were significant risk factors for ICAS (*P* < 0.05). The percentages of ICAS in hypertension and diabetics were 56.9% and 22.8%, respectively. Subjects with ICAS had higher SBP, DBP, and fasting glucose levels than these without ICAS. However, there was no significant difference regarding sex, body mass index, waistline, smoking, alcohol habit, fasting total cholesterol (TCHO), and triglycerides (TG) between ICAS or non-ICAS groups. Besides, there were 25% of subjects had a smoking habit and 19.6% had an alcohol habit in the ECAS group, higher than those in the non-ECAS group. Male subjects were more susceptible to ECAS than female subjects (*P* < 0.05), and persons with higher TCHO (*P* < 0.05) but not TG were more likely to develop ECAS.

**Table 2:** Univariate analysis of risk factors for ICAS and ECAS

		Intracranial artery			Extracranial artery		
		Without ICAS	ICAS	p	Without ECAS	ECAS	p
Age	2598	55 ± 11	61 ± 11	<0.001	52 ± 10	63 ± 10	<0.001
Male, n(%)	2598	1033 (41.7)	51 (41.5)	0.95	664 (36.6)	420 (53.6)	<0.001
BMI (kg/m2)	2598	25.5 ± 3.9	25.9 ± 3.5	0.24	25.5 ± 3.9	25.6 ± 3.9	0.78
Waistline (cm)	2598	84.1 ± 11.1	85.3 ± 10.9	0.24	84.1 ± 10.8	84.4 ± 11.6	0.50
HTN, n(%)	2598	611 (24.7)	70 (56.9)	<0.001	376 (20.7)	305 (28.6)	<0.001
DM, n(%)	2598	169 (6.8)	28 (22.8)	<0.001	108 (6.0)	89 (11.4)	<0.001
Smoking habit, n(%)	2598	480 (19.4)	31 (25.2)	0.11	315 (17.4)	196 (25.0)	<0.001
Alcohol intake, n(%)	2598	397 (16.0)	18 (14.3)	0.68	261 (14.4)	154 (19.6)	0.001
Mean SBP (mmHg)	2598	129 ± 19	137 ± 22	<0.001	127 ± 18	136 ± 21	<0.001
Mean DBP (mmHg)	2598	81 ± 10	84 ± 10	0.003	81 ± 10	83 ± 10	<0.001
Fasting glucose (mmol/l)	2598	5.5 ± 1.8	6.0 ± 2.4	0.04	5.5 ± 1.9	5.7 ± 1.9	0.002
TCHO (mmol/l)	2598	5.5 ± 1.5	5.5 ± 1.8	0.82	5.4 ± 1.6	5.6 ± 1.5	0.005
TG (mmol/l)	2598	1.7 ± 1.2	1.7 ± 1.0	0.72	1.8 ± 1.2	1.8 ± 1.3	0.82
Creatine (umol/l)	2598	79.8 ± 23	79.4 ± 23	0.86	79.3 ± 23.0	80.9 ± 23.0	0.12

*ICAS* intracranial artery stenosis, *ECAS* extracranial artery stenosis, *BMI* body mass index, *HTN* hypertension, *DM* diabetes mellitus, *SBP* systolic blood pressure, *DBP* diastolic blood pressure, *TCHO* total cholesterol, *TG* total triglyceride

Based on logistic regression analyses, as shown in Table 3, three factors emerged as independent risk factors (*P* < 0.05) of ICAS: age (95% confidence interval [CI] = 1.01–1.04, odds ratio [OR] = 1.07), HTN (95% CI = 1.98–4.37, OR = 2.94), and DM (95% CI = 1.72–4.38, OR = 2.75). For ECAS, Table 4 shows that five factors emerged as independent risk factors (*P* < 0.05): age (95% CI = 1.09–1.11, OR = 1.10), DM (95% CI = 1.10–2.12, OR = 1.53), male sex (95% CI = 1.01–1.02, OR = 1.01), SBP (95% CI = 1.95–2.88, OR = 2.37), and TCHO (95% CI = 1.00–1.13, OR = 1.06).

**Table 3:** Multi-variate analysis of risk factors for ICAS

	OR	95% CI	p
Age	1.07	1.01-1.04	0.008
HTN	2.94	1.98-4.37	<0.001
DM	2.75	1.72-4.38	<0.001

*ICAS* intracranial artery stenosis, *OR* odds ratio, *CI* confidence interval, *HTN* hypertension, *DM* diabetes mellitus

**Table 4:** Multi-variate analysis of risk factors for ECAS

	OR	95% CI	p
Age	1.10	1.09-1.11	<0.001
DM	1.53	1.10-2.12	0.01
Male	1.01	1.01-1.02	<0.001
SBP	2.37	1.95-2.88	<0.001
TCHO	1.06	1.00-1.13	0.04

*ECAS* extracranial artery stenosis, *OR* odds ratio, CI confidence interval, *DM* diabetes mellitus, *SBP* systolic blood pressure, *TCHO* total cholesterol

## Discussion

Many of the studies on ICAS and ECAS of Chinese population reported previously were mostly urban hospital-based investigations while the exact rural situations were still unknown [14, 15]. As a local study focusing on both ICAS and ECAS of the Chinese pure rural population, our findings have international significance because of the high volume of rural population in China and the relatively high prevalence of ICAS in Chinese population. The residents lived in the same village for generations, and shared the same lifestyle, so the results were less influenced by different daily habits of subjects. In our study the prevalence of ICAS in rural areas was lower than what reported by Bae et al., namely 24.5% for Korean asymptomatic intracranial stenosis [16]. With similar TCD method, Cancio et al. [17] reported 8.6% prevalence of ICAS in Spanish asymptomatic intracranial atherosclerosis of city communities. The reason of relatively lower prevalence of ICAS in our study was likely to be the difference of ethnicity and urban and rural differences with pure rural villagers in our study. Our study found that 3% of the population had intracranial stenosis in anterior circulation, which was lower than the 5.9% of MCA stenosis in asymptomatic residents in Guangdong province, which was reported by Huang [14]. The difference may be due to age variability: the mean age is 55 yr. in our study and 64 yr. in Huang’s study.

As for the prevalence of ECAS, the epidemiology study was scarce in China. A study from Spain pointed out that, the prevalence of stenosis reached 30.3% for stenosis <50% and 6.1% for stenosis ≥50% in males aged 75-84 [18]. Asians, Hispanics, and Africans are more prone to ICAS while Caucasian individuals are more prone to ECAS [19–21]. Thus, striking difference existed in the distribution of atherosclerotic stenosis in the cerebral vasculature among different races.

In the context of primary prevention, it is important to identify factors related to the presence of ECAS and ICAS in a stroke-free individual. In our risk-factor analysis, age, HTN, and DM were independent risk factors for ICAS while age, male sex, DM, SBP, and TCHOL were independent risk factors for ECAS. The findings of independent risk factors for ICAS were compatible with those of Cancio et al. [17] who carried out studies in Caucasians, and Hee-Joon et al. [16] who studied a Korean population. Meta-analysis has shown that female sex is a risk factor for asymptomatic ICAS [22]. However, our study found that ICAS had no significant correlation with sex, conversely male were prone to be ECAS, which could be explained by the fact that men tended to have habits of smoking, alcohol use, and a high calorie and fat intake [23]. Our study found that TCHOL was a major risk factor for ECAS, consistent with the findings of Bang et al. [24]. Other traditional risk factors, including smoking and hyperlipidemia [25], were not found significantly associated with cerebrovascular atherosclerosis in our study.

In our study, hypertension appeared as the most relevant risk factor for ICAS but not for ECAS, different from the work by Su et al., a community-based study in Taiwan indicating hypertension strongly influenced ECAS [26]. Although it had been suggested that HTN was a more important risk factor for intracranial small vessel atherosclerosis, in this study, a positive correlation was found in a multivariate analysis between ECAS and SBP, not in those with a history of HTN. This result was consistent with that of a previous study [27]. Epidemiological studies [28, 29] have demonstrated the importance of SBP as a risk factor for cerebral infarction. Another possible reason was that subjects in this population have a lower education level and are unaware of the HTN incidence. Documentation of HTN was collected by self-report rather than a clinical follow-up, which may lead to some bias. Therefore, it could not be definitively concluded that ECAS was not related to HTN. DM, a major component of metabolic syndrome, is an established risk factor for ECAS [30]. However, its role in ICAS is yet to be elucidated, and previous studies have yielded controversial results [16, 31]. Our study indicated that DM was a significant predictor of both asymptomatic ECAS and ICAS, which has been supported by studies on symptomatic individuals [32, 33].

Although ICAS and ECAS shared similar risk factors, differences still existed between them. A possible explanation was that different structures and hemodynamics of extra- and intracranial arteries may cause different response to pathophysiological processes and various components in the blood [34, 35]. These findings provide an insight into the pathogenesis of intra- and extracranial atherosclerosis.

There were comments for our study. Unlike previous studies evaluating only MCA stenosis, we detected all intracerebral vascular arteries including the ACA and terminals of ICA, MCA, PCA, and VA as well as BA. In addition, all of the examinations were performed by trained technicians under a standard protocol. The results were sound and informative. There were some limitations in our study. First, as it was a cross-sectional study, there are some limitations in the evaluation of the real impact of risk factors in the development of ICAS and ECAS. Second, TCD may be less accurate than angiography. However, it was well suited for screening a large number of subjects with high negative predictive values for ICAS. Third, as the population in our study was largely composed of female subjects because most male villagers left home and went to work in the city, a substantial selection bias was possible. Fourth, subjects with poor acoustic windows were excluded from the analysis, which may lead to an underestimation of the prevalence in stroke-free subjects. Fifth, all the risk factors reported in our study were associated with atherosclerosis which accounted mostly for cerebral artery stenosis but not all, due to few other causes, including arteritis, infection, and other immunological related disorders, et al. We did not apply criteria for exclusion of other causes which may affect the purity of the study.

## Conclusion

In conclusion, this epidemiological survey of asymptomatic pure rural populations in northern China found ICAS more common than ECAS with stenosis ≥50%. Age, HTN, and DM were significant risk factors for ICAS. Male sex and TCHO were more related to ECAS rather than ICAS. In spite of a local study, due to the large rural population in China, the effective prevention and treatment of cerebral vascular diseases was not only beneficial to China but to the whole world. These results may contribute to further understanding of cerebrovascular stenosis in rural China, facilitate the international fellows to perform better international clinical research and provide further insight into the prevention of these diseases.

## Abbreviations

ACA: Anterior cerebral artery
CCAs: Common carotid arteries
DM: Diabetes mellitus
ECAS: Extracranial carotid artery stenosis
HTN: Hypertension
ICA: Internal carotid artery
ICAS: Intracranial carotid artery stenosis
IMT: Intima media thickness
MCA: Middle cerebral artery
PCA: Posterior cerebral artery
SBP: Systolic blood pressure
TCD: Transcranial Doppler ultrasonography
TCHO: Total cholesterol
